# Choanoflagellates alongside diverse uncultured predatory protists consume the abundant open-ocean cyanobacterium *Prochlorococcus*

**DOI:** 10.1073/pnas.2302388120

**Published:** 2023-06-26

**Authors:** Susanne Wilken, Charmaine C. M. Yung, Camille Poirier, Ramon Massana, Valeria Jimenez, Alexandra Z. Worden

**Affiliations:** ^a^Department of Ocean Sciences, University of California Santa Cruz, Santa Cruz, CA 95064; ^b^Department of Marine Biology and Oceanography, Institut de Ciències del Mar (ICM-CSIC), 08003 Barcelona, Spain; ^c^Department of Freshwater and Marine Ecology, Institute for Biodiversity and Ecosystem Dynamics, University of Amsterdam, 1090 GE Amsterdam, Netherlands; ^d^Ocean Ecosystems Biology, GEOMAR Helmholtz Centre for Ocean Research, 24105 Kiel, Germany; ^e^Marine Biological Laboratory, Woods Hole, MA 02543

**Keywords:** microbial food webs, trophic transfer, heterotrophic nanoflagellates, choanoflagellates, *picocyanobacteria*

## Abstract

*Prochlorococcus* is a key member of open-ocean primary producer communities. Despite its importance, little is known about the predators that consume this cyanobacterium and make its biomass available to higher trophic levels. We identify potential predators along a gradient wherein *Prochlorococcus* abundance increased from near detection limits (coastal California) to >200,000 cells mL^−1^ (subtropical North Pacific Gyre). A replicated RNA-Stable Isotope Probing experiment involving the in situ community, and labeled *Prochlorococcus* as prey, revealed choanoflagellates as the most active predators of *Prochlorococcus*, alongside a radiolarian, chrysophytes, dictyochophytes, and specific MAST lineages. These predators were not appropriately highlighted in multiyear conventional 18S rRNA gene amplicon surveys where dinoflagellates and other taxa had highest relative amplicon abundances across the gradient. In identifying direct consumers of *Prochlorococcus*, we reveal food-web linkages of individual protistan taxa and resolve routes of carbon transfer from the base of marine food webs.

Marine primary production roughly equals that of terrestrial ecosystems but differs in being performed by unicellular phytoplankton with high turnover rates (1). Globally, about two-thirds of marine primary production is thought to be rapidly consumed by predatory protists (2), which hence play critical roles in regulating phytoplankton and carbon flux (3). Abundances of *Prochlorococcus*, the dominant open-ocean phytoplankter, are controlled through tight coupling between growth and mortality (4, 5). However, the key predators consuming *Prochlorococcus* remain largely unknown. Knowledge regarding specific interactions and ecological roles of heterotrophic predatory protists has been hindered by their diversity (3, 6), cultivation difficulties (7), and sparsity of in situ studies at ecologically and evolutionarily relevant taxonomic levels (8–10). Thus, roles of predatory protists are often excluded or highly simplified in ecosystem models, which is problematic given the importance of trophic transfer from abundant primary producers, like *Prochlorococcus*, and how it may transition in future oceans. Here, we use culture independent methods to examine predatory protists in the Pacific Ocean and expose specific lineages that actively consume *Prochlorococcus*.

## Results and Discussion

**Fig. 1.**
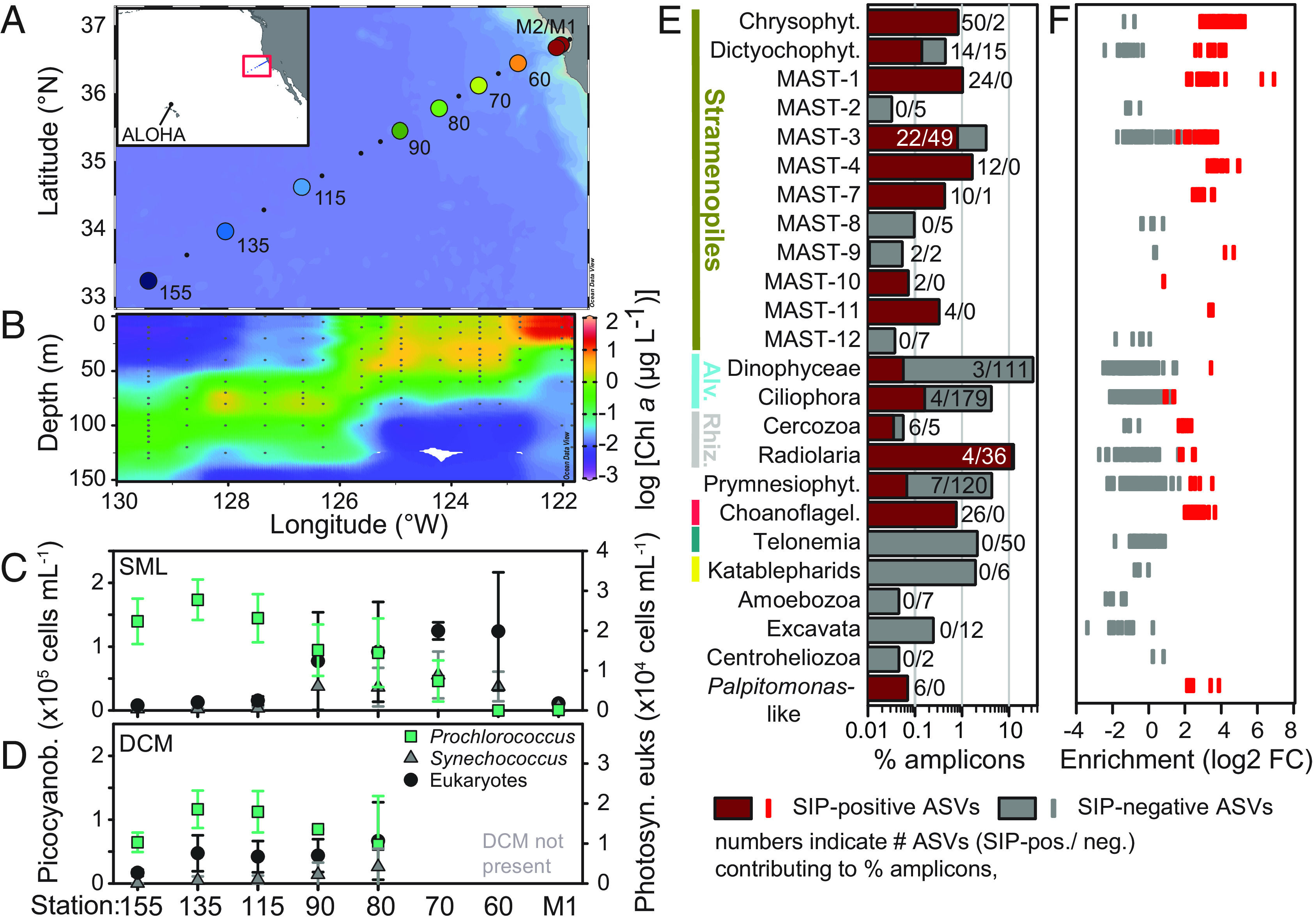
Phytoplankton prey communities from the coast to open ocean and predatory protists feeding on *Prochlorococcus*. (*A*) Stations sampled and overall Pacific region (*Inset*). (*B*) Chlorophyll *a* concentrations (2009 only for simplicity) show the development of a DCM offshore. (*C* and *D*) Mean cell abundances (±SD) of *Prochlorococcus, Synechococcus*, and eukaryotic picophytoplankton in the (*C*) Surface Mixed Layer (SML) and (*D*) DCM (if present), generally from 6-y annual sampling. Transect mid-region data are variable due to interannual shifts (13). (*E*) Relative contributions of protistan groups identified as feeding (maroon) and those not feeding on *Prochlorococcus* (gray) to total 18S-V9 rRNA amplicons in the RNA-SIP experiment at 67-80. Note: log scale. (*F*) Relative enrichment in amplicon abundance in heavy fractions of the density gradient due to isotope incorporation in ^13^C/^15^N-labeled treatments compared to the same fractions from the controls (^12^C/^14^N). Shown is the average enrichment across all five heavy density windows per ASV (see also Dataset S2).

Multiple years of sampling from coastal California into oligotrophic waters of the North Pacific Subtropical Gyre (NPSG) established the progressive development of nutrient-poor surface waters, a deep chlorophyll maximum (DCM), and transitions in picophytoplankton cell abundances (Fig. 1 *A*–*D* and Dataset S1). As observed previously (4), *Prochlorococcus* becomes numerically dominant offshore, while its abundance decreases shoreward as the cyanobacterium *Synechococcus* and eukaryotic picophytoplankton increase. *Prochlorococcus* reached 2 × 10^5^ cells mL^−1^ at the western-most stations, comparable to maxima in the NPSG and North Atlantic Subtropical Gyre (11–13).

To characterize predators actively consuming *Prochlorococcus*, we performed an in situ experiment using RNA-stable isotope probing (RNA-SIP), involving amendment of natural communities with ^13^C/^15^N-labeled *Prochlorococcus* cells and, separately, controls with unlabeled (^12^C/^14^N) cells, each in biological triplicate, followed by 18S-V9 rRNA amplicon analyses. The 227 protistan predators (ASV-level discrimination, Dataset S2) captured feeding on *Prochlorococcus* collectively caused grazing mortality to balance *Prochlorococcus* growth and included uncultivated stramenopiles, such as MArine STramenopiles (MAST)-1, -4 and -7 already known to consume cyanobacteria (8, 9), chrysophytes, some dictyochophytes, and a few prymnesiophytes (Fig. 1*E*). The latter three groups may include predatory mixotrophs (if they retain plastids), a functional group increasingly recognized as *Prochlorococcus* consumers (10, 14), although when and where they feed is less clear. Here, we ascertain that they were actively feeding but cannot predict their capability to photosynthesize, due to the limitations of sequences for making this delineation without characterized isolates. Strong enrichment in heavy density gradient fractions indicated high predation on *Prochlorococcus* by several predators (Fig. 1*F*); however, for those with lower enrichment, it remains unclear whether they consumed less *Prochlorococcus* or utilized other unlabeled prey organisms that diluted the isotopic signal gained from consumed *Prochlorococcus* cells.

The RNA-SIP approach to tracking prey into predators also identified a number of protists not known to consume *Prochlorococcus* or phytoplankton in general. These included ASVs related to the deep-branching cryptistan predator *Palpitomonas* (6), the cercozoan *Minorisa,* the uncultivated Radiolarian lineage RAD-B (15), and uncultivated MAST-10, MAST-11, as well as MAST-3. Strikingly, every choanoflagellate detected had consumed *Prochlorococcus*. This was surprising because choanoflagellates are rarely mentioned in current surveys on this realm of the oceanic food web, despite being recognized bacterivores in coastal habitats. This is partially because many studies have focused on their surface-attached feeding mechanisms or developmental biology (16, 17) and because choanoflagellates do not stand out in relative amplicon abundance surveys (18). Yet three decades ago, precision microscopy in several pelagic environments demonstrated that loricate choanoflagellates comprised 25 to 30% of all heterotrophic nanoflagellates (HNF), as termed in the earlier marine literature that investigated microbial predation by microscopy (17). Additionally, *Synechococcus* was noted in choanoflagellate food vacuoles (nonquantitative data). Here, the choanoflagellates consuming *Prochlorococcus* were primarily from the Stephanoecidae family. These loricate choanoflagellates were thought to be coastal; however, recently, small oceanic forms have been reported throughout warm oceans, connecting well with our results (19). Historically, as taxonomic insights improved through molecular analyses, contributions of MAST to HNF were emphasized (7). We posit Stephanoecidae choanoflagellates comprise an important portion of the active HNF community, but that prior sampling procedures disrupted their lorica, the structure used for identification (17, 19).

**Fig. 2.**
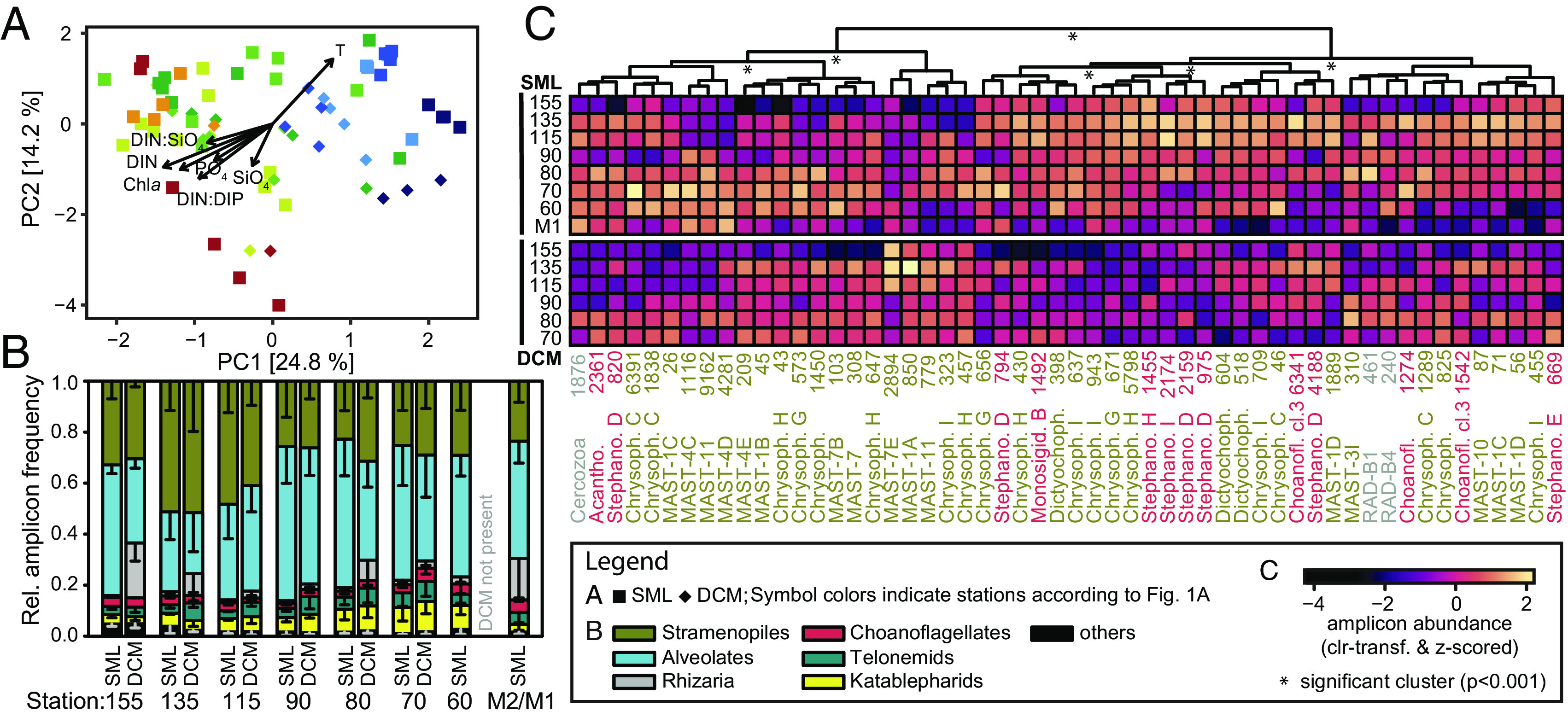
Predatory protist distribution patterns. (*A*) Ordination of predatory heterotrophic protist communities by principal component analysis, overlain with vectors of environmental parameters. Coastal and open-ocean habitats differed in community composition (PERMANOVA: F_6_ = 4.61, *P* = 0.001), as did the SML and DCM (PERMANOVA: F_1_ = 4.86, *P* = 0.001). (*B*) Relative abundance contributions of all protists putatively functioning as heterotrophic predators collapsed to broad groups, based on 18S-V4 amplicon analyses. (*C*) Habitat distributions of RNA-SIP-identified *Prochlorococcus* predators from the open-ocean shoreward. Shown are 18S-V4 amplicon abundances averaged across years with Aitchison distance and Ward-linkage based hierarchical clustering of ASVs. Information following taxon names indicates membership in known phylogenetic clades followed by amplicon identifiers (Dataset S1). All dictyochophytes were Pedinellales.

To assess the distribution of *Prochlorococcus* predators across the environmental gradient, we examined protistan communities using 18S-V4 rRNA gene profiling. Predatory heterotrophic protists contributed a relatively consistent fraction of the total 18S rRNA gene amplicon abundances, varying between 10 to 20% in the surface mixed layer (SML) and 9 to 14% in the DCM (where present), but were compositionally different across the transect (Fig. 2*A* and Dataset S1). Because nutritional modes vary even between closely related taxa, likely mixotrophic predators were excluded as they could not be accurately distinguished from purely photosynthetic species if uncultured. Alveolates and stramenopiles showed high relative amplicon abundances, followed by telonemids and katablepharids, while choanoflagellates appeared relatively rare (Fig. 2*B*). Within stramenopiles, MAST-3 showed highest relative amplicon abundance, especially in offshore surface waters, while MAST-4 appeared more active and abundant based on the RNA-SIP experiment, but not based on survey relative abundances. An important caveat is that relative amplicon abundance trends do not reflect organismal abundances, given 18S rRNA gene copy number variations between taxa. Overall, most active predators identified by RNA-SIP did not stand out in transect relative amplicon compositional patterns; thus, the survey patterns did not appropriately highlight the protists actively feeding on *Prochlorococcus* that were responsible for trophic transfer of its carbon.

We next focused on the distribution of only those taxa identified as actively feeding by RNA-SIP. Hierarchical clustering of predator distributions showed that most choanoflagellates had highest relative amplicon abundances in nutrient-poor offshore stations (Fig. 2*C*). Other predators clustering here were mixotrophic Pedinellales (dictyochophytes), which perhaps represent a more oligotrophic counterpart to the Florenciellales dictyochophytes (14). Florenciellales isolates from near Hawaii consume *Prochlorococcus* and are present in mesotrophic waters, but at, e.g., BATS, are only seen during more nutrient-rich winter periods (13). Coastal stations and the DCM had relatively more stramenopile predators, including MAST-4 and -7. These stations have low *Prochlorococcus* abundances but have other small phytoplankton (Fig. 1 *C* and *D*) and bacteria (13), suggesting consumption of a broad prey spectrum alongside *Prochlorococcus*.

In conclusion, we expose predatory protists actively feeding on *Prochlorococcus*, most of which do not appear of marked importance based on conventional amplicon surveys. Moreover, loricate choanoflagellates, Pedinellales dictyochophytes, MAST-3, -10 and -11, and radiolarian lineage Rad-B have never been observed to feed on *Prochlorococcus*. The diversity of predators identified experimentally herein likely represent just a subset of the predators that *Prochlorococcus* might face in the ocean. These divergent eukaryotes utilize varied ecological strategies and distinct feeding mechanisms and are likely controlled by different factors, driving disparate predator–prey dynamics, and potential coevolution of predator and prey. Further disentanglement of ecological differentiation should be possible with named predators. This will be essential for predicting trophic control of primary producers and resolving the multiple alternative routes through food webs along which *Prochlorococcus* biomass might sustain higher trophic levels.

## Materials and Methods

During six fall (September to October) cruises along line 67 (4), samples were collected for nutrient, chlorophyll, flow cytometry, and 18S-V4 rRNA gene analyses, alongside temperature and salinity data. After taxonomic assignment, amplicons were partitioned based on putative nutritional strategies, specifically photosynthetic (i.e., plastid-bearing), parasitic, or heterotrophic predatory (*SI Appendix*). RNA-SIP was performed in 2015, by addition of ^13^C/^15^N-labeled *Prochlorococcus* MED4 [HLI ecotype present in the Pacific (11)] to biological triplicates of the natural community at station 67-80; triplicated controls used unlabeled MED4. Predators incorporating isotopes from *Prochlorococcus* were identified by density gradient centrifugation, followed by further analysis of 15 density fractions per sample via cDNA-synthesis and 18S-V9 rRNA gene sequencing. Grazing mortality was quantified by a two-step dilution assay. See *SI Appendix* for further details. Transect cruise and RNA-SIP experiment data are available in Datasets S1 and S2, respectively.

## Supplementary Material

Appendix 01 (PDF)

Dataset S01 (XLSX)

Dataset S02 (XLSX)

## Data Availability

Amplicons have been deposited in Sequence Read Archive (SRA) under bioproject PRJNA972531. All other data are included in the article and/or supporting information.
