# Supplementary material for: Choanoflagellates alongside diverse uncultured predatory protists consume the abundant open-ocean cyanobacterium Prochlorococcus
Source: Proc Natl Acad Sci U S A. 2023 Jun 26;120(27):e2302388120. doi: 10.1073/pnas.2302388120 (PMC10318953; doi:10.1073/pnas.2302388120)
Supplement: Supplementary file 1 — Appendix 01 (PDF) [file pnas.2302388120.sapp.pdf]

PNAS

Supporting Information for
Choanoflagellates alongside diverse uncultured predatory protists consume the abundant open-ocean cyanobacterium *Prochlorococcus*

Susanne Wilken, Charmaine C.M. Yung, Camille Poirier, Ramon Massana, Valeria Jimenez, Alexandra Z. Worden

Susanne Wilken, Alexandra Z. Worden
Email: s.wilken@uva.nl, azworden@mbl.edu

This PDF file includes:

Supporting text
Legends for Datasets S1 to S2
SI References

Other supporting materials for this manuscript include the following:

Datasets S1 to S2

Supporting Information Text

Extended Materials and Methods

Field sampling along CalCOFI line 67

Field surveys were conducted along the California Cooperative Oceanographic Fisheries Initiative (CalCOFI) line 67, stretching from the central Californian coast southwestwards into the Pacific Ocean (1). Cruises aboard the RV Western Flyer were performed in the fall of 2009, 2011, 2012, 2013, 2015 and 2016. Sample metadata are summarized in dataset S1. Water was collected in 10L Niskin bottles attached to a conductivity-temperature-depth (CTD) rosette sampler (Sea-Bird Electronics).

Concentrations of chlorophyll *a*, as well as the nutrients nitrate, nitrite, ammonium, phosphate, and silicate were determined as previously described (2). Phytoplankton cellular abundances were enumerated via flow cytometry as previously described (3). For this, samples were fixed with a final concentration of 0.25% glutaraldehyde, incubated at room temperature for 20 min, and flash frozen in liquid nitrogen prior to storage at -80°C. Analysis was performed on a BD Influx flow cytometer equipped with a 488 nm laser at a flow rate of 25 µL min^{-1}. Phytoplankton populations were identified based on their forward angle light scatter, chlorophyll-*a* autofluorescence (i.e., fluorescence detected in the 692/40 nm bandpass filter) and phycoerythrin autofluorescence for *Synechococcus* (detected in the 572/27 nm bandpass filter).

For community profiling of microbial eukaryotes based on amplicon sequencing of the 18S rRNA gene, 1L water samples were filtered onto Supor membrane filters (PALL, 0.2 µm pore size, 47 mm diameter). Filters were stored at -80°C prior to DNA extraction using the DNeasy plant kit (Qiagen) with the addition of a bead-beating step (4).

RNA-stable isotope probing experiment

Preparation of isotope labeled Prochlorococcus

Prochlorococcus MED4 was grown axenically in Pro99 medium (5) in an artificial seawater (ASW) base and incubated at 21°C and 100 µmol photons m^{-2} s^{-1} under a 14h:10h light-dark cycle. Axenicity was confirmed prior to the experiment by filtering a culture aliquot onto a polycarbonate filter (0.2 µm pore size, 25 mm diameter), staining with DAPI, and observation by epifluorescence microscopy. Additionally, a culture aliquot was transferred into a bacterial test medium, incubated in darkness, and checked for visually observable turbidity after 14 days. The stock culture was split into two aliquots, one for growth on stable isotope (^{13}C-bicarbonate and ^{15}N-ammonium) containing medium and one for growth under control conditions with the respective light isotopes (^{12}C-bicarbonate and ^{14}N-ammonium). Both media were prepared from the same batch of artificial seawater without the addition of bicarbonate. Sterile filtered bicarbonate and ammonium chloride stock solutions consisting of light or heavy isotopes depending on treatment were added after autoclaving. Stock cultures were split into two volumes of 50 mL, centrifuged at 8000 g for 10 min and resuspended in either stable-isotope or control medium. Cultures were maintained in their respective medium for 4 days, and immediately prior to their use in the experiment, cells were washed three times by gravity filtration over Supor membrane filters (PALL, 0.2 µm pore size, 47 mm diameter) and resuspension in ASW.

Experimental incubations and sampling

An RNA stable isotope probing (RNA-SIP) experiment was performed at station 67-80 on 15th September 2015, in which the planktonic microbial community was amended with ^{13}C and ^{15}N-

labeled *Prochlorococcus* as prey to track predation on *Prochlorococcus* by protistan predators. Water for experimental incubations was collected from 5 m depth and prefiltered through a 70 µm mesh to remove larger predators. Experimental treatments were performed in triplicate and included (i) a stable-isotope prey addition treatment with ^{13}C and ^{15}N-labeled *Prochlorococcus* added to a final abundance of 5.8 x 10^5 cells mL^{-1}, (ii) a prey-addition control with the unlabeled *Prochlorococcus* cells added to natural seawater as above, (iii) a prey only control with *Prochlorococcus* added to cell-free (0.2 µm filtered) seawater in order to test for potential mortality of *Prochlorococcus* due to other reasons than grazing, and finally two additional control treatments to confirm grazing mortality of natural *Prochlorococcus* populations, which consist of (iv) a dilution treatment with 20% natural seawater diluted with 80% cell-free seawater (0.2 µm filtered) and (v) undiluted natural seawater. Each treatment was performed in biological triplicate, except the prey only control, which was performed in duplicate. Incubations were performed in 4 L polycarbonate bottles placed in an on-deck incubator shielded to in-situ light conditions with neutral density foil (LEE) and kept at in-situ temperature maintained by a continuous flow-through of seawater. Incubations were started after sunset and lasted 24 h.

Flow cytometry samples were taken from each incubation bottle at the start and end of the experiment, fixed with 0.25% glutaraldehyde, incubated for 20 min in the dark, flash-frozen, and stored at -80°C until analysis as described above. Net and gross growth rates of natural *Prochlorococcus* populations, as well as their grazing mortality, were quantified from the two-step dilution treatments (6), and confirmed gross growth rates of *Prochlorococcus* to be balanced by similar rates of mortality due to grazing (gross growth rate of 0.25±0.03 (SE) d^{-1} and grazing mortality of 0.21±0.04 (SE) d^{-1}). Furthermore, the added *Prochlorococcus* survived under the experimental conditions with zero net growth in the whole, unfiltered seawater and a growth rate of 0.19 d^{-1} in filtered seawater.

Samples for quantification of ^{13}C in dissolved inorganic carbon (DIC) were taken to control for potential remineralization, which could lead to indirect routes of label incorporation by non-predatory protists. To this end, water samples were filtered through 0.2 µm pore size syringe-top filters and stored at 4 °C in crimp-sealed glass vials prior to analysis at the Stable Isotope Facility at the University of California, Davis. While a slight ^{13}C-enrichment was measurable after 24 h of incubation in the treatment with isotope-labeled *Prochlorococcus* ($\delta^{13}C_{VPDB}$ of 4.88±0.10 (SE) ‰) relative to the natural community at the start of the experiment ($\delta^{13}C_{VPDB}$ of 1.59±0.09 (SE) ‰), these values correspond to 1.125 and 1.129 % of ^{13}C in the DIC pool, respectively, and thus are insufficient to cause the much larger isotope ratio required for separation of labeled RNA in the density gradient centrifugation.

Nucleic acid samples were taken from the bulk 70 µm pre-filtered seawater during set-up of the experiment and from each bottle individually after incubation. 1 L samples were filtered onto Supor membrane filters (PALL, 0.2 µm pore size, 47 mm diameter) and stored at -80°C.

RNA extraction, fractionation, and cDNA-synthesis

RNA was extracted using the TotallyRNA kit (Life Technologies, Grand Island, NY, USA). Cell lysis was achieved by transferring the filters with 1 mL of lysis buffer into 2 mL screw cap tubes pre-filled with ~200 µL of a 1:1 mixture by volume of 0.1 mm and 0.5 mm diameter autoclaved glass beads (Biospec Products, Bartlesville, OK, USA). After two minutes of bead beating, filters and lysis buffer were transferred into a 15 mL screw cap tube containing an additional 4 mL lysis buffer. Samples were vortexed for 1 min, and centrifuged for 3 min at 10,000g and 4°C. The lysate was transferred to a new 15 mL tube and extraction proceeded according to the manufacturer's guidelines. DNA was digested using the TurboDNA-free kit (Life Technologies) following the manufacturer's instructions. RNA integrity was evaluated on a Bioanalyzer (Agilent, Santa Clara, CA, USA) and

quantity was determined on a Qubit fluorometer (Life Technologies). RNA yield ranged from 0.8 to 1.4 µg.

RNA fractionation was performed as described earlier (7). Briefly, depending on RNA-yield per sample, between 0.8 and 1.1 µg of RNA were separated in a CsTFA gradient using 5.2 mL polyallomer tubes and centrifugation in an Optima XE-90 ultracentrifuge (Becton-Dickinson) equipped with a VTi90 rotor at 20°C and 45000 rpm for 64 h. Density gradients were fractionated into 20 fractions of 255 µL that were collected from below by piercing a hole into the bottom of the tube and displacing the gradient solution by water from above using a needle and syringe pump (KD Scientific, LEGATO 185) operated at a flow rate of 4 µL s^{-1}. Densities were determined for each fraction using 50 µL of sample for measurement on a digital refractometer (Reichert, AR200). In the remaining sample, RNA was precipitated by adding two volumes of ice-cold isopropanol, incubating at -20°C for at least 2 h, and centrifuging at 14,000 g and 4°C for 20 min. Pellets were washed in another 150 µL of isopropanol, centrifuged again at 14,000 g and 4°C for 5 min, dried after removal of the supernatant and resuspended in 10 µL water. RNA concentrations were then quantified on a Qubit fluorometer with the high sensitivity RNA kit using 3 µL of each RNA-fraction. Synthesis of cDNA was performed using 6 µL of each RNA-fraction as template with the Super Script III First-Strand Synthesis SuperMix (Invitrogen) and random hexamer priming following manufacturer guidelines. Resulting cDNA was quantified using the Qubit ssDNA kit with negative control cDNA synthesis-reactions as blanks.

PCR-amplification, sequencing, and sequence analysis

PCR-reactions targeting the V9 hypervariable region of the 18S rRNA gene were performed using the Illumina-adapted versions of primers 1389f and 1510r (8). 50 µL reaction volume contained up to 5 ng of the template depending on cDNA-synthesis yield, 0.2 µM of each primer, 0.3 mM dNTPs, 1.6 mM $MgSO_4$, and 0.2 µL High Fidelity Platinum Taq (Invitrogen). Thermocycling consisted of 95°C for 2 min, 32 cycles of 94°C for 15 s, 56°C for 30 s, and 68°C for 60 s, followed by a final elongation step at 68°C for 7 min.

After paired-end (PE) library sequencing (300bp) on an Illumina MiSeq instrument (Illumina, San Diego, CA, USA) sequences were demultiplexed and assigned to corresponding samples using CASAVA (Illumina). Low-quality sequence ends were trimmed at Phred quality (Q) of 25 using a 10 bp running window using Sickle 1.33 (9). Paired-end reads were merged using USEARCH v.9.0.2132 (10) when reads had a ≥ 40 bp overlap with maximum 5% mismatch. The merged reads were then filtered to remove reads with maximum error rate > 0.001. Only sequences with exact matches to both primers were kept and primer sequences were trimmed using Cutadapt (11) v.1.13. Sequences were analyzed as amplicon sequence variants (ASVs) based on USEARCH-UNOISE (12), and taxonomy was assigned using classify-sklearn (13) by searching against both the Protist Ribosomal Reference database v.4.9.2 (14) and SILVA SSU rRNA (15) databases (versions 4.9.2 and 132, respectively). Assignments were checked for consensus for the SIP-positive taxa. Conflicting assignments were found for six ASVs assigned as Chytridiomycota by PR2, but as *Palpitomonas* by SILVA. Using these ASVs as queries in a BLAST search against the NCBI database resulted in hits of 82-85% identity that included mainly cryptophytes and katablepharids, and occasionally other taxa such as cercozoa, telonemids and prasinophytes, but never chytrids or other opisthokonts. This does not support an assignment as Chytridiomycota, but is consistent with a relation to *Palpitomonas* as a basal cryptist lineage (16).

Statistical analysis of isotope incorporation

For determining isotope incorporation, multiple window high-resolution stable isotope probing (MW-HR-SIP) has been performed as described earlier (17), but accounting for the biological replication in our experiment. To test for the enrichment of sequences in the heavy fractions of the stable-

isotope treatment relative to the control treatment, six density windows were defined across 15 of the buoyant density fractions, one ‘light’ control window (1.745-1.760 g mL^{-1}) containing the peak of unlabeled RNA found in the control treatment, and five ‘heavy’ windows spanning higher buoyant densities (1.76-1.79 g mL^{-1}) that overlapped with each other, but not with the control window. Only ASVs that were present in at least 50% of the fractions were included in the analysis. Isotope enrichment was then tested using DESeq2 (18) with a model containing buoyant window, replicate (to perform paired tests between control and treatment replicate gradients formed in the same centrifuge run), and treatment (control versus stable isotope addition) as factors. Additionally, an interaction term between buoyant window and treatment was included to allow testing for enrichment in the heavy windows of the isotope treatment in particular. An ASV was defined as incorporating stable isotopes, if significantly enriched (log2 fold change > 0.5, and one-sided Wald test with $p < 0.05$) in at least two of the heavy buoyant density windows of the isotope treatment relative to the respective windows in the control treatment (and after correction for potential differences among treatments in the ’light’ control window).

Protistan community composition along the CalCOFI line 67 transect

PCR and Illumina sequencing

For community analysis the V4 hypervariable region of the 18S rRNA gene was amplified from whole community DNA samples using the Illumina-adapted TAReuk454FWD1 and TAReukREV3 primers (19). PCR reactions contained 10 ng of template DNA and 1X 5 Prime HotMasterMix (Quanta Biosciences, Beverly, MA, United States) as well as 0.4 mg mL^{-1} BSA (NEB) and 0.4 µM of each primer. PCR reactions were amplified using the following protocol: 94 °C for 3 min; and 30 cycles at 94 °C for 45 s, 50 °C for 60 s and 72 °C for 90 s; with a final extension at 72 °C for 10 min. Triplicate reactions per sample were pooled. Paired-end (PE) library sequencing (PE 2 x 300bp) was performed on an Illumina MiSeq instrument (Illumina, San Diego, CA, USA).

Sequences were demultiplexed, quality trimmed, and analyzed as described above. However, after trimming of primer sequences, singleton sequences were excluded and the remaining sequences were *de novo* clustered at 99% similarity by UPARSE (20). This step was necessary, because samples from six years of field sampling had been sequenced on several sequencing runs, and the ASV-level discrimination as performed for RNA-SIP samples appeared to be impacted by the specific run in which a sample had been sequenced. Furthermore, 99% OTU clustering of the longer and more variable V4 region still provides taxonomic resolution comparable to that of the V9 amplicons. The most abundant sequence of each operational taxonomic unit (OTU) was picked for classification using classify-sklearn (13) by searching against the Protist Ribosomal Reference database v.4.9.2 (14).

Classification of protistan nutritional modes

To analyze the community composition of presumably predatory protists, only sequences from unicellular eukaryotes were retained, removing all sequences assigned to metazoans, streptophytes, multicellular algal lineages, or sequences assigned at a taxonomic level insufficient to distinguish between unicellular and multicellular lineages. Next, OTUs were classified by their nutrition based on their taxonomic assignment, similar as done before (21). First, we distinguished between plastidic protists containing their own photosynthetic plastids and non-plastidic, heterotrophic protists. Already this first step is prone to error, as some groups such as dinoflagellates and chrysophytes contain photosynthetic and unpigmented heterotrophic species that are closely related to each other, and this distinction is thus impossible to confidently make based on a relatively rough taxonomic assignment of environmental sequences. Because the classification becomes even more complicated, if considering mixotrophic protists (combining a

photosynthetic with a predatory nutrition) for which the predatory potential is difficult to prove, we did not attempt to distinguish plastid containing protists further into purely photosynthetic and mixotrophic ones. Among non-plastidic protists, we then further distinguished between acquired phototrophs that harbor photosynthetic endosymbionts or kleptoplastids, parasitic protists, and free-living heterotrophic protists. For the free-living heterotrophic protists, a predatory lifestyle was assumed in the absence of more specific information, such as known osmo- or saprotrophic lifestyles.

This resulted in the majority of amplicons belonging to protists classified as either plastidic or parasitic (see Dataset S1), with plastidic protists being relatively more important in coastal waters (62.5 ± 10.9 % of amplicons close to shore compared to 9.4 ± 0.4 % and 7.8 ± 1.1 % in the mixed layer and DCM at the outermost station 67-155), and parasitic taxa having higher relative amplicon abundance in oligotrophic offshore waters (60.3 ± 3.7 % of amplicons in the mixed layer of station 67-135, versus 19.6 ± 8.5 % close to shore). Predatory heterotrophic protists constituted a more stable percentage of total amplicon abundance (9.6-19.2 % in the mixed layer and 8.7-13.3 % in the DCM) with only small percentages of acquired phototrophs (0.2-6.9%) and osmo- or saprotrophs (0.05-0.44%). Additionally, some non-plastidic protists (2.1-12.7% of amplicons) could not be assigned to any more specific nutritional mode based on their taxonomic assignment, while for others (4.9-13.4% of amplicons) the assigned taxonomic level was too coarse to even infer the presence or absence of a plastid. These latter were not assigned any nutritional mode (NA).

Following the classification into nutritional modes, the taxonomic composition of predatory heterotrophic protists was analyzed further as representing potential predators of *Prochlorococcus*. This excludes mixotrophic predators which are increasingly recognized as important bacterivores and in some cases also feed on *Prochlorococcus* (22, 23). As mentioned above, reliable identification of mixotrophic taxa still relies on cultured isolates (23), and inferring a mixotrophic lifestyle from environmental sequences is thus difficult.

Statistical analysis of community composition

For multivariate analysis of the entire protistan community, or the subset of only the predatory heterotrophic population, data were first filtered to retain only taxa with a relative abundance of at least 0.05% and presence in at least 10% of the samples before dividing the dataset based on the nutritional categorization. For the subset of predatory heterotrophs, principal component analysis was performed using Aitchison distance (Euclidian distance after centered log-ratio transformation) as recommended in (24) using the R-packages phyloseq (25) and vegan (26). Environmental variables were log-transformed if non-normally distributed and fitted to the ordination using the 'envfit' function in vegan. A preliminary analysis showed nitrate and ammonium concentrations to be closely aligned and salinity to be non-significant. We therefore excluded salinity from the final analysis and combined nitrate, nitrite and ammonium into the single variable dissolved inorganic nitrogen (DIN). The variables temperature, chlorophyll a, silicate, phosphate, DIN, as well as the ratios of DIN to silicate, and DIN to dissolved inorganic phosphorus (DIP) were then fitted to the ordination. Finally, differences in community composition between stations and between the surface mixed layer versus DCM were tested by PERMANOVA in vegan.

Distribution patterns of Prochlorococcus *predators*

In order to analyze the distribution of predators identified as feeding on *Prochlorococcus* in the RNA-SIP experiment across the CalCOFI-line 67 transect, we had to connect the 18S-V9 ASVs used to identify predators in the SIP-experiment to the 18S-V4 OTUs used in the field survey. For this we selected only those taxonomic groups for which all 18S-V9 ASVs assigned to the respective group were identified as actively feeding based in the SIP experiment. We then selected all 18S-V4 OTUs from the survey dataset that were assigned to the same taxonomic group and had been

detected in the SIP-experimental community to represent these SIP-positive predators. Because MAST-3 contained 22 SIP-positive ASVs, but all of them fell into subclades that also contained SIP-negative ASVs, we performed an additional step by blasting both 18S-V4 and 18S-V9 sequences against the NCBI database. This resulted in one additional 18S-V4 OTU (OTU310, MAST-3I) that could be linked to the SIP-positive 18S-V9 ASV 381, based on hits (100%ID for V9 and 99.5%ID for V4) to the same sequence. Finally, to further assess the distribution of these predators their centered log-ratio transformed and z-scored amplicon abundances across the transect (averaged over sampling years) were clustered by hierarchical clustering based on Euclidian distance and Ward linkage. Significance of the resulting clusters was tested using the statistical Significance for Hierarchical Clustering (SHC) approach implemented in the R-package SigClust2 (27).

Dataset S1 (separate file). Data from the field survey cruises contain A) metadata and environmental parameters measured for each sample, B) the full 18S V4 rDNA amplicon dataset with reads per OTU and sample, taxonomic assignments, nutritional categorization, and V4 sequences, and C) relative amplicon abundances of different nutritional types along the survey transect (synthesized from B).

Dataset S2 (separate file). Data from the RNA-SIP experiment contain A) metadata from the buoyant density gradient fractions including treatment, replicate, fractions number and density; B) the 18S V9 rRNA amplicon dataset with reads per ASV and fraction, taxonomic assignments, and V9 sequences; C) statistical results (log2 fold change and adjusted p-value) for each comparison of reads retrieved per ASV in the buoyant density windows 2 to 6 relative to the control window 1.

SI References

1. S.J. Bograd, D.A. Checkley, W.S. Wooster, CalCOFI – a half century of physical, chemical, and biological research in the California Current System. *Deep Sea Res Part II* **50**, 2349-2353 (2003).
2. T. Pennington, F. Chavez, Seasonal fluctuations of temperature, salinity, nitrate, chlorophyll and primary production at station H3/M1 over 1989–1996 in Monterey Bay, California. *Deep Sea Res Part II* **47**, 947-974 (2000).
3. M. Cuvelier *et al.*, Targeted metagenomics and ecology of globally important uncultured eukaryotic phytoplankton. *Proc. Natl. Acad. Sci. U.S.A.* **107**, 14679-14684 (2010).
4. E. Demir-Hilton *et al.*, Global distribution patterns of distinct clades of the photosynthetic picoeukaryote *Ostreococcus*. *ISME J.* **6**, 481-492 (2011).
5. L. Moore *et al.*, Culturing the marine cyanobacterium *Prochlorococcus*. *Limnol. Oceanogr. Methods* **5**, 353-362 (2007).
6. A.Z. Worden, B.J. Binder, Application of dilution experiments for measuring growth and mortality rates among *Prochlorococcus* and *Synechococcus* populations in oligotrophic environments. *Aquat. Microb. Ecol.* **30**, 159-174 (2003).
7. A.S. Whiteley, B. Thomson, T. Lueders, M. Manefield, RNA Stable-Isotope Probing. *Nat. Protoc.* **2**, 838-844 (2007).
8. C. De Vargas *et al.*, Eukaryotic plankton diversity in the sunlit ocean. *Science* **348**, 1261605 (2015).
9. N.A. Joshi, J.N. Fass, Sickle: A sliding-window, adaptive, quality-based trimming tool for FastQ files [software]. (2011).
10. R.C. Edgar, Search and clustering orders of magnitude faster than BLAST. *Bioinformatics* **26**, 2460-2461 (2010).

11. M. Martin, Cutadapt removes adapter sequences from high-throughput sequencing reads. *EMBnet J*. **17**, 10-12 (2011).
12. R.C. Edgar, H. Flyvbjerg, Error filtering, pair assembly and error correction for next-generation sequencing reads. *Bioinformatics* **31**, 3476–3482 (2015).
13. F. Pedregosa *et al.*, Scikit-learn: Machine Learning in Python. *J Mach Learn Res.* **12**, 2825–2830 (2011).
14. L. Guillou *et al.*, The Protist Ribosomal Reference database (PR2): a catalog of unicellular eukaryote small sub-unit rRNA sequences with curated taxonomy. *Nucleic Acids Res.* **41**, D597-604 (2013).
15. C. Quast *et al.*, The SILVA ribosomal RNA gene database project: improved data processing and web-based tools. *Nucl. Acids Res.* **41**, D590-D596 (2013).
16. A. Yabuki et al., *Palpitomonas bilix* represents a basal cryptist lineage – insights into the character evolution in Cryptista. *Sci. Rep.* **4**, 4641 (2014).
17. N.D. Youngblut, S.E. Barnett, D.H. Buckley, HTSSIP: An R package for analysis of high throughput sequencing data from nucleic acid stable isotope probing (SIP) experiments. *PLoS One* **13**, e0189616 (2018).
18. M.I. Love, W. Hubert, S. Anders, Moderated estimation of fold change and dispersion for RNA-Seq data with DESeq2. *Genome Biol.* **15**, 550 (2012).
19. T. Stoeck *et al.*, Multiple marker parallel tag environmental DNA sequencing reveals a highly complex eukaryotic community in marine anoxic water. *Mol. Ecol.* **19**, 21-31 (2010).
20. R.C. Edgar, UPARSE: Highly accurate OTU sequences from microbial amplicon reads. *Nat. Methods* **10**, 996-998 (2013).
21. M. Hamilton *et al.*, Spatiotemporal variations in Antarctic protistan cmmunities highlight phytoplankton diversity and seasonal dominance by a novel Cryptophyte lineage. *mBio* **12**, e02973-21 (2021).
22. M. Hartmann, M.V. Zubkov, D.J. Scanlan, C. Lepère, In situ interactions between photosynthetic picoeukaryotes and bacterioplankton in the Atlantic Ocean: evidence for mixotrophy. *Environ. Microbiol.* **5**, 835-840 (2013).
23. Q. Li, K.F. Edwards, C.R. Schvarcz, G.F. Steward, Broad phylogenetic and functional diversity among mixotrophic consumers of *Prochlorococcus*. *ISME J.* **16**, 1557-1569 (2022).
24. G.B. Gloor, J.M. Macklaim, V. Pawlowsky-Glahn, J.J. Egozcue, Microbiome datasets are compositional – and this is not optional. *Front. Microbiol.* **8**, 2224 (2017).
25. P.J. McMurdie, S. Holmes, Phyloseq - a bioconductor package for handling and analysis of high-throughput phylogenetic sequence data. *Pac. Symp. Biocomput.* **2012**, 235-246 (2012).
26. P. Dixon. VEGAN, a package of R functions for community ecology. *J. Veg. Sci.* **14**, 927-930 (2003).
27. P.K. Kimes, Y. Liu, D.H. Hayes, J.S. Marron, Statistical significance for hierarchical clustering. *Biometrics* **73**, 811-821 (2017).
